# Hypothalamic primary cilium: A hub for metabolic homeostasis

**DOI:** 10.1038/s12276-021-00644-5

**Published:** 2021-07-01

**Authors:** Dong Joo Yang, Jessica Hong, Ki Woo Kim

**Affiliations:** 1 Departments of Oral Biology and Applied Biological Science, BK21 Four, Yonsei University College of Dentistry, Seoul, 03722 Korea; 2 Brown University, Providence, RI 02912 USA

**Keywords:** Homeostasis, Hypothalamus

## Abstract

Obesity is a global health problem that is associated with adverse consequences such as the development of metabolic disorders, including cardiovascular disease, neurodegenerative disorders, and type 2 diabetes. A major cause of obesity is metabolic imbalance, which results from insufficient physical activity and excess energy intake. Understanding the pathogenesis of obesity, as well as other metabolic disorders, is important in the development of methods for prevention and therapy. The coordination of energy balance takes place in the hypothalamus, a major brain region that maintains body homeostasis. The primary cilium is an organelle that has recently received attention because of its role in controlling energy balance in the hypothalamus. Defects in proteins required for ciliary function and formation, both in humans and in mice, have been shown to cause various metabolic disorders. In this review, we provide an overview of the critical functions of primary cilia, particularly in hypothalamic areas, and briefly summarize the studies on the primary roles of cilia in specific neurons relating to metabolic homeostasis.

## Metabolic disease: Nerve cell cilia in the hypothalamus

Hair-like sensory structures called primary cilia that protrude from cells in the brain’s hypothalamus are becoming implicated in the control of the body’s energy balance. Defects in proteins needed for the cilia to form and function have been shown to cause several metabolic disorders. Primary cilia are found in nearly all mammalian cells and researchers in South Korea and the USA, led by Ki Woo Kim of Yonsei University in Seoul, have reviewed their functions, particularly those in the hypothalamus. They summarize research into the involvement of cilia on specific nerve cells in maintaining energy balance. They conclude that further studies of hypothalamic primary cilia offer great potential to understand the role of the central nervous system in energy balance and possibly provide new ways to prevent and treat obesity and other metabolic disorders.

## Introduction

**Fig. 1:**
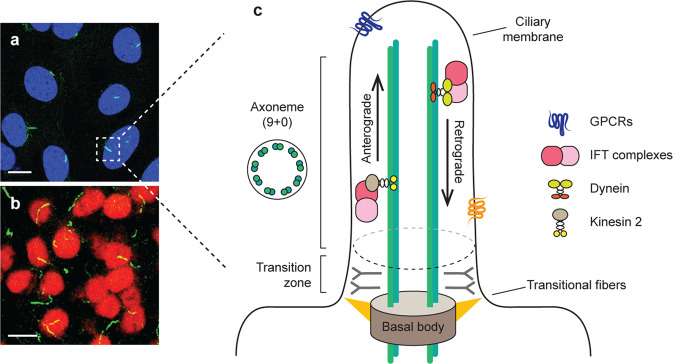
Schematic structure of primary cilia. Immunofluorescence images of primary cilia (green, ADCY3) in hypothalamic cells (**a**) and arcuate nuclei (**b**). Scale = 20 μm. Schematic structure of primary cilia (**c**). The primary cilium is an antenna-like organelle that receives diverse signals from the extracellular environment. It is comprised of the ciliary membrane surrounding the microtubule-based axoneme. The nine parallel microtubule doublets of the axoneme, which show “9 + 0” rings, form the backbone of the appendage, while the basal body acts as a microtubule-organizing center. The components that are transported from the basal body to the ciliary tip by anterograde transport rely on the intraflagellar transport (IFT) protein attached to the motor protein kinesin 2. In contrast, retrograde transport from the ciliary tip to the cytoplasm depends on dynein motor proteins. The ciliary membrane is highly enriched with several receptors, including G protein-coupled receptors (GPCRs).

The cilium is a hair-like organelle formed with cell membrane and is present on nearly every mammalian cell. Cilia have historically been classified as either motile or immotile. Structurally, a cilium consists of a microtubule-based axoneme covered by a ciliary membrane (Fig. 1). The axoneme emerges from the basal body, a centriole-derived and microtubule-organizing center, extending from the cell surface into the extracellular space [1]. In terms of their formation and functions, cilia are constructed through a microtubule motor-based transport system that consists of intraflagellar transport (IFT) complexes that bind directly to cargos and their motors kinesin-2 and dynein, enabling travel across the axoneme [2, 3]. The IFT complex consists of two distinct subcomplexes, complexes A and B. Complex A is needed for retrograde movement from the ciliary tip to the cytoplasm, while complex B is utilized in anterograde transport from the cellular base to the ciliary tip. Balanced transport systems are important for ciliogenesis, as faulty regulation of these factors causes abnormal cilia formation [3].

Most mammalian cells have a single immotile cilium, called the primary cilium, which has evolved to receive various signals from extracellular stimuli [1, 4]. Due to their ability to detect sensory cues, primary cilia are considered cellular antennae. However, little attention had been directed to the importance of primary cilia until the discovery of their association with polycystic kidney disease (PKD) [5]. PKD is an inherited genetic disorder that results in progressive renal cyst formation due to abnormal primary cilia function. Later, the medical significance of primary cilia, beyond their relation to PKD, became increasingly evident, with evidence showing that the structural and functional anomalies of primary cilia arise from genetic mutations in ciliary proteins. These proteins are closely related to human diseases comprehensively called ciliopathies, including retinal degeneration, polydactyly, hypertension, and obesity [6]. While it has been established that primary cilia have critical and diverse functions in several cell types, many questions regarding the precise functions of primary cilia in these cell types, particularly neuronal primary cilia, remain.

Body weight regulation has garnered significant attention due to its importance in maintaining energy balance, which is primarily determined by food intake and energy expenditure. Research on feeding behavior and energy consumption is needed to cure metabolic diseases such as obesity and diabetes, along with their complications. The brain, particularly the hypothalamus, plays a critical role in integrating and coordinating several types of signals, including hormones and nutrients, to maintain body homeostasis. Recently, primary cilia have received attention because of their functioning as sensory centers in controlling energy balance. Early evidence linking primary cilia and energy homeostasis was realized upon the discovery that proteins associated with human obesity syndromes, such as Alström and Bardet-Biedl syndromes, localize to primary cilia. Mutations in these proteins, both in humans and in mice, result in organisms displaying severe obesity and diabetes [6–8]. In 2007, Davenport et al. directly assessed the importance of primary cilia by utilizing conditional knockout (KO) of ciliogenesis genes (*Tg737* and *Kif3a*). This study was the first to show that neuronal primary cilia are required for normal energy homeostasis [9]. Since then, neuronal primary cilia have emerged as critical organelles in the integration of the complex signals in metabolic homeostasis [10–12]. Therefore, in this review, we summarize the evidence supporting the role of hypothalamic primary cilia in controlling metabolic homeostasis.

## Main text

### The hypothalamus, a site for energy balance control

**Fig. 2:**
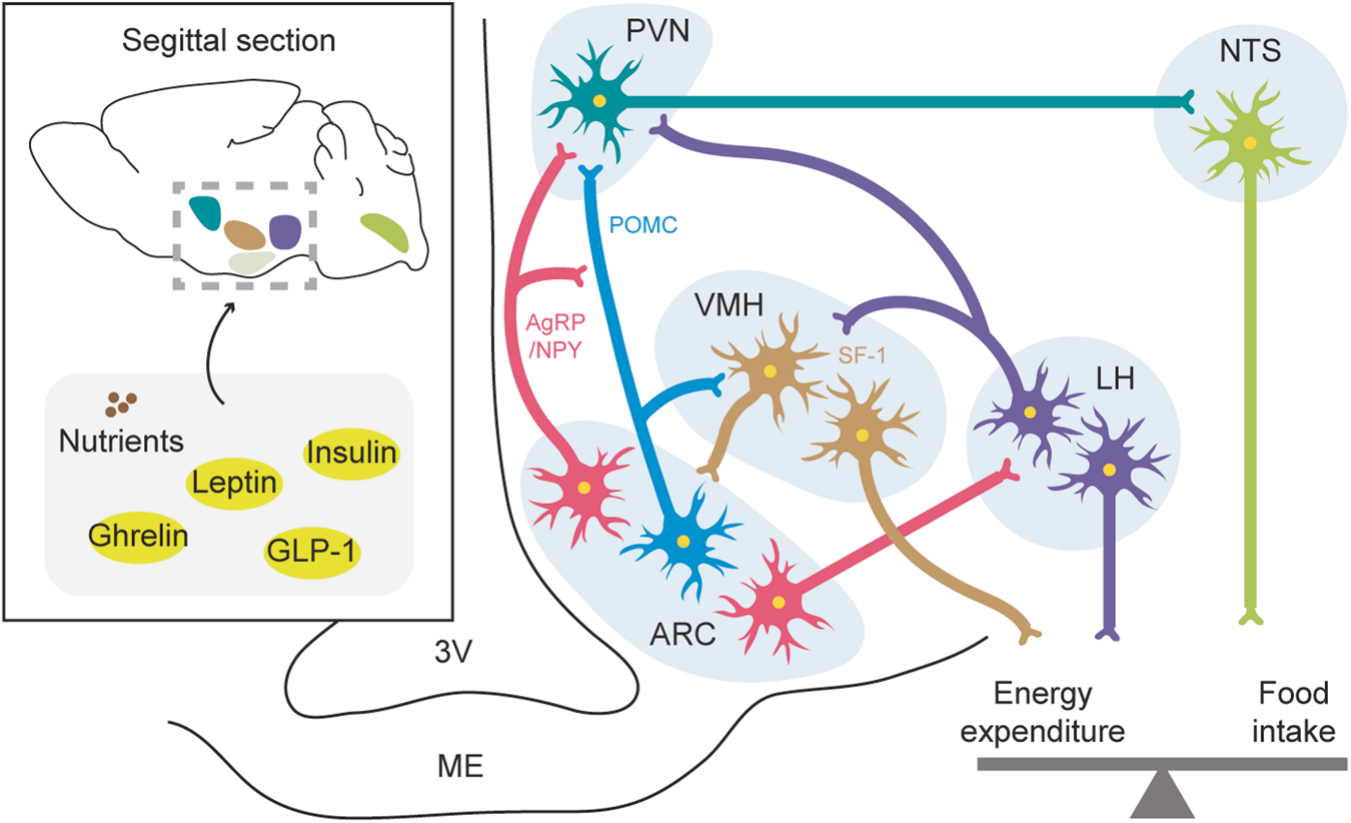
Hypothalamic nuclei involved in the regulation of energy balance. Energy homeostasis is regulated by a complex feedback loop involving endocrine and neuronal signals originating from peripheral organs and intrahypothalamic communications. The ARC is a key nucleus that houses POMC and AgRP/NPY neurons, which integrate the aforementioned signals. These neurons project to various nuclei, including the PVN, VMH, and LH. In turn, the ARC receives input from the VMH and LH. The NTS receives projections from the ARC, PVN, VMH, and LH and regulates multiple metabolic effectors of energy balance. 3 V, third ventricle; AgRP, agouti-related protein; ARC, arcuate nucleus; LH, lateral hypothalamus; ME, median eminence; NPY, neuropeptide Y; NTS, nucleus of tractus solitarius; POMC, proopiomelanocortin; PVN, paraventricular nucleus; SF-1, steroidogenic factor 1; and VMH, ventromedial hypothalamus.

The hypothalamus is a key brain region in the balance of body homeostasis [13, 14]. It encompasses several anatomically well-defined nuclei, including the arcuate nucleus (ARC), as well as the ventromedial hypothalamus (VMH), dorsomedial hypothalamus (DMH), lateral hypothalamus (LH), and paraventricular nucleus (PVN) of the hypothalamus (Fig. 2) [15]. The ARC, which is located at the base of the hypothalamus and is in close proximity to the median eminence (ME), primarily senses metabolic signals from the periphery via systemic circulation. There are two distinct functionally antagonistic neurons: (i) orexigenic agouti-related peptide (AgRP)- and neuropeptide Y (NPY)-expressing neurons and (ii) anorexigenic proopiomelanocortin (POMC)-expressing neurons [16, 17]. These neuronal populations are called first-order neurons because they integrate peripheral, nutritional, and hormonal cues to control energy balance [18, 19] through various hormone receptors distributed over the neuronal membrane, such as leptin receptor (LepR) and insulin receptor (IR) [20]. For example, leptin binds to receptors expressed at the surface of POMC and AgRP neurons. Once leptin binds to LepR, the neurons are either activated or inhibited and regulate food intake and energy expenditure by releasing melanocortin peptides, which are key products that control energy balance. POMC neurons produce α-melanocyte-stimulating hormone (α-MSH), an agonist of melanocortin receptor 4 (MC4R), which is key in regulating energy and glucose homeostasis. In contrast, the inverse agonist AgRP suppresses MC4R activity and simultaneously antagonizes the effects of α-MSH [18, 21, 22]. These neurons project to both intrahypothalamic neurons (e.g., neurons in the VMH, DMH, LH, and PVN) and extrahypothalamic neurons (e.g., the nucleus of tractus solitarius (NTS) and the mesolimbic reward system), communicating information regarding peripheral energy availability to other brain areas (Fig. 2) [23]. Among these areas, the PVN seems to be the center of the melanocortin system. Once either AgRP or α-MSH binds to MC4R in the neurons of the PVN, feeding behavior is modulated, possibly through the autonomic nervous system [24–29].

The PVN lies alongside the top portion of the third ventricle (3 V) in the anterior hypothalamus, and it plays an imperative role in the regulation of energy balance and endocrinological activities. Lesions in the PVN produce obesity and hyperphagia via control of the sympathetic outflow to peripheral metabolic organs [30, 31]. PVN neurons also constitute the primary endocrine control center, synthesizing and secreting neuropeptides that have a net catabolic action, such as corticotropin-releasing hormone (CRH) [32] and thyrotropin-releasing hormone (TRH) [33].

The VMH is an oval-shaped hypothalamic nucleus located directly above the ARC. This brain area contains neurons that sense glucose and leptin and thus is the site controlling the regulation of body weight and glucose homeostasis [34, 35]. A number of studies have shown that steroidogenic factor-1 (SF-1) neurons in the VMH actively participate in insulin and leptin signaling in energy expenditure and glucose homeostasis, exerting minor effects on feeding behavior [36–38]. In addition, VMH neurons can modulate sympathetic nerve activity (SNA), which is thought to underlie a variety of neuronal mechanisms in the VMH [37, 39–43]. For instance, electrical stimulation of the VMH induces glycogenolysis and gluconeogenesis in the liver and increases blood pressure and heart rate through sympathoexcitation [44, 45], whereas destruction of the VMH by bilateral lesions causes hyperphagia, obesity, hyperglycemia, and reduced SNA [14, 46, 47]. POMC neurons in the ARC project to the VMH and control food intake via stimulation of MC4R and subsequent activation of brain-derived neurotrophic factor (BDNF) [48]. On the other hand, the VMH, which consists mostly of glutamatergic neurons, projects to the ARC and transduces excitatory input to both POMC and AgRP neurons [49].

The LH is important in receiving sensory signals from the periphery, including the gut and liver [50, 51]. This brain region contains large numbers of glucose-receptive neurons that respond to circulating glucose levels, most likely via pathways ascending from the hypothalamus [52]. In contrast to the PVN and VMH, bilateral destruction of the lateral portion of the LH abolishes food intake, thus resulting in weight loss in rats, even in those who had previously been induced to obesity [14]. Therefore, the LH is considered a feeding center in the hypothalamus.

Overall, the diverse neuronal networks of various hypothalamic regions are instrumental in regulating energy balance, each individually functioning to maintain body homeostasis.

### Neuronal primary cilia and regulation of body homeostasis

While it has been known for decades that primary cilia exist in neurons, their precise functions in each type of neuron remain poorly understood. In the past 20 years, the importance of neuronal cilia has been reported through various studies, and the evidence for this importance is increasing daily. An early observation showing a correlation between metabolic disorders and neuronal primary cilia function was based on conditional tamoxifen-inducible KO of the core ciliogenesis gene *Kif3a* and the intraflagellar transport 88 (*Ift88*, also called *Tg737*) protein [9]. Germline ablation of either *Kif3a* or *Tg737* in adult mice resulted in hyperphagia-induced obesity with elevated serum leptin, insulin, and glucose levels. Restricting dietary intake prevented increases in both body weight and serum hormones, supporting the notion that the obesity and diabetic phenotypes were a consequence of hyperphagic behaviors. Interestingly, specific deletion of *Kif3a* or *Tg737* using synapsin 1-cre (Syn1-cre) led to similar results of obesity and leptin resistance, indicating that neuronal primary cilia may play important roles in the regulation of body weight homeostasis [9].

Adenylyl cyclase 3 (ADCY3) catalyzes the synthesis of cyclic AMP (cAMP), an important second messenger in signaling pathways, from ATP [53, 54]. In the central nervous system (CNS), cAMP plays a critical role in neuronal functions, including survival, growth, differentiation, and synaptogenesis [55]. In 2007, neuronal cilia were found to be specifically enriched with ADCY3 [56], and it has subsequently been shown that global *Adcy3*-KO mice exhibit adult onset obesity due to disruption of cAMP signaling in the hypothalamus [57]. In addition, a number of G protein-coupled receptors (GPCRs), including somatostatin receptor 3 (SSTR3), serotonin receptor 6 (5-HT6R), melanin-concentrating hormone receptor 1 (MCHR1), and more recently MC4R, have been reported to be located in neuronal cilia [58]. Once ciliary components are defective, aberrant ciliary localization and signaling of certain GPCRs are displayed, causing obesity.

Overall, these results indicate that both the structural and functional roles of neuronal primary cilia are necessary to control energy homeostasis. Indeed, several recent pieces of evidence suggest that hypothalamic cilia may function together as a metabolic signaling center, which is critical to the control of body homeostasis (Table 1) [9, 41, 59–62].

**Table 1:** Metabolic phenotypes of hypothalamic primary cilia dysfunction.

Gene	Mutation	Target area	Metabolic phenotype	Ref.
*Structural defect*				
Ift88 (Tg737)	CAGG-cre^ER^::Ift88^loxP^	Global KO	Hyperphagic-induced obesity, hyperleptinemia, hyperinsulinemia, hyperglycemia	[9]
	Syn1-cre::Ift88^loxP^	Neurons	Hyperphagia-induced obesity, hyperleptinemia, hyperglycemia	[9, 64]
	POMC-cre::Ift88^loxP^	POMC neurons		
	POMC-cre^ER^::Ift88^loxP^		No change	[64]
	LepRb-cre::Ift88^loxP^	LepRb-expressing neurons	Mild obesity, decreased energy expenditure, minimal leptin resistance	[62]
	SF1-cre::Ift88^loxP^	SF-1 neurons	Obesity, decreased energy expenditure, leptin resistance, hyperinsulinemia, hyperglycemia, high bone density	[41]
Kif3a	CAGG-cre^ER^::Kif3a^loxP^	Global KO	Hyperphagic-induced obesity, hyperleptinemia, hyperinsulinemia, hyperglycemia	[9, 64]
	POMC-cre::Kif3a^loxP^	POMC neurons		
*Functional defect*				
Adcy3	Adcy3^null/null^	Global KO	Obesity, insulin resistance	[57]
	AAV-Cre::Adcy3^loxP^	VMH	Obesity, hyperphagia	[77] [62]
Bbs1	Nestin-cre::Bbs1^loxP^	CNS	Obesity, hyperphagia	
	LepRb-cre::Bbs1^loxP^	LepRb-expressing neurons	Obesity, hyperphagia, decreased energy expenditure, leptin resistance	
	POMC-cre::Bbs1^loxP^	POMC neurons	Obesity, hyperphagia, hyperglycemia, insulin resistance	[70]
	AgRP-cre::Bbs1^loxP^	AgRP neurons	Obesity, hyperinsulinemia	
Bbs2, Bbs4, Bbs6	Bbs2^null/null^, Bbs4^null/null^, and Bbs6 ^null/null^	Global KO	Obesity, hyperphagia, hypertension, leptin resistance	[69]
Mc4r	SIM1-cre::Mc4r^pPro230Leu^	Sim1 neurons	Obesity	[75]
GPR88	SIM1-cre::AAV-GPR88*	Sim1 neurons	Obesity	

*G protein-coupled receptor 88

### Primary cilia in the arcuate nucleus

Primary cilia in both orexigenic and anorexigenic neurons in the ARC have been implicated not only in the regulation of food intake but also in responses to hormones, including leptin and insulin. Systemic ablation of ciliary genes from neurons using Syn1-cre led to hyperphagic-induced obesity, and the obesity phenotype of both *Kif3a-*Syn1-KO and *Tg737-*Syn1-KO mice was reproduced in POMC-specific *Kif3a*-KO (Kif3a-Pomc-KO) mice [9]. Both male and female Kif3a-Pomc-KO mice exhibited significant increases in weight, primarily due to hyperphagia. Deletion of *Kif3a* in POMC neurons also led to elevated levels of leptin and insulin, indicating that cilia in POMC neurons are required to maintain both energy balance and responses to satiety signals such as leptin and insulin signals. Furthermore, introduction of short inhibitory RNA (siRNA) targeting *Kif3a* or *Ift88* in the ARC led to an increase in food intake and a decrease in energy expenditure, manifesting the obese phenotype [63].

A recent study showed that inhibition of ciliogenesis in developing POMC neurons, which was realized by depleting *Kif3a* or *Ift88*, led to adulthood obesity in mice [64]; these mice showed disruption of axonal projections through impaired lysosomal protein degradation in POMC neurons. In contrast, ciliary deletion in adult POMC neurons using tamoxifen-inducible cre did not lead to significant changes in body weight, fat mass, or lean mass, suggesting that primary cilia in adult POMC neurons have a minimal role in the regulation of energy balance.

Bardet-Biedl syndrome (BBS) is a rare recessive genetic disease, and its patients commonly display severe obesity [7, 8]. The BBS protein complex, a subset of the IFT complex, also participates in the transportation of ciliary membrane proteins [65, 66]. In contrast to *Ift88* and *Kif3a*, loss-of-function mutations in BBS genes do not lead to complete structural defects of primary cilia; however, they disrupt ciliary functions [67]. Germline ablation of the *Bbs2* and *Bbs4* (*Bbs2*^−/−^ and *Bbs4*^−/−^) genes led to hyperphagia-induced obesity, coupled with reduced phosphor-STAT3 levels in the hypothalamus. Furthermore, research has shown that a lack of localization of SSTR3 and MCHR1 to cilia in *Bbs2*^−/−^ and *Bbs4*^−/−^ mouse neurons [68], indicating that the altered signaling caused by mislocalization of ciliary signaling proteins underlies the BBS phenotypes. Subsequently, *Bbs2*^−/−^, *Bbs4*^−/−^, and *Bbs6*^−/−^ mutant mice were reported to display hyperleptinemia as a result of defective leptin signaling [61, 69]. Molecular studies revealed that Bbs1, a component of the BBSome, directly bound to the leptin receptor long-form (LepRb) and participated in LepRb trafficking. Consistently, *Bbs1* M390R, which is the most common mutant of Bbs1 found in patients, showed decreased interaction with LepRb, implying that the LepRb trafficking and subsequent signaling pathways may be altered in BBS mutant patients. These studies suggest that ciliary BBS proteins might be required for normal leptin signaling and thus for the maintenance of energy balance [61]. Later, the results of studies on targeted disruption of the BBSome by deletion of the *Bbs1* gene in the brain using Nestin-cre (Nestin^Cre^/Bbs1^fl/fl^) showed obesity in mice, and the obese phenotype was reproduced by ablation of *Bbs1* in LepRb-expressing neurons, LRb^Cre^/Bbs1^fl/fl62^. Both Nestin^Cre^/Bbs1^fl/fl^ and LRb^Cre^/Bbs1^fl/fl^ mice demonstrated increased food intake coupled with reduced hypothalamic *Pomc* levels. On the other hand, disruption of *Ift88* in LepRb-expressing cells caused minimal gains in body weight and fat mass. In contrast to LRb^Cre^/Bbs1^fl/fl^, no difference in food intake was found in LRb^Cre^/Ift88^fl/fl^ mice; however, this model showed a decrease in both energy expenditure and body temperature. Moreover, mechanistic studies demonstrated that the deletion of the expression of BBS proteins, but not *Ift88*, impaired LepRb trafficking to the plasma membrane, leading to central leptin resistance in a manner independent of obesity. In summary, these results demonstrated that cilium-mutant mouse models may display obesity through different and independent mechanisms.

Recent studies from the same group that performed the LepRb trafficking study reported the role of *Bbs1* in POMC and AgRP neurons. Deletion of *Bbs1* in either POMC or AgRP neurons led to result similar to those obtained with models of leptin-signaling deficiency [70]. Both POMC^Cre^/Bbs1^fl/fl^ and AgRP^Cre^/Bbs1^fl/fl^ mice showed obesity associated with an increase in food intake. In addition, by lacking the BBSome protein, as identified by the impairment of serotonin receptor 5-HT_2C_R, it was shown that trafficking to the membrane contributed to the hypothalamic BBSome control of energy balance and handling of metabolic receptors [70].

### Abnormal functions of primary cilia in the paraventricular nucleus cause obesity

MC4R is a central component of the melanocortin system, a hypothalamic network that integrates information from the periphery and regulates food intake and energy expenditure [71]. Mutations in *Mc4r* are the most common monogenic cause of severe obesity in humans, as well as in rodents [72–74]. Although the expression pattern of MC4R has been well documented, previous attempts to determine the subcellular localization of MC4R in vivo have been unsuccessful. In 2018, it was first reported that MC4R localizes to primary cilia in a subset of mouse hypothalamic neurons, including the PVN, where it colocalized with ADCY3 [75]. This finding was particularly relevant since it was previously reported that ADCY3 mutations are closely associated with human obesity, and *Adcy3*-KO mice exhibited obesity upon disruption of cAMP signaling in the hypothalamus [57]. In this study, it was discovered that the impaired localization of MC4R in primary cilia caused MC4R mutations, which led to suppression of ADCY3 activity in cilia and, in turn, the acquisition of the obese phenotype in mice [75]. Furthermore, specific inhibition of ADCY3 through the use of GPR88, a constitutively active version of the cilia-specific Gα_i_-protein-coupled receptor, resulted in suppression of cAMP production and, consequently, increased body weight. These findings suggest that MC4R and ADCY3 may positively regulate cAMP generation in neuronal primary cilia of the PVN, where MC4R is highly expressed, and impaired cAMP signaling in the primary cilia of MC4R-expressing neurons leads to obesity. However, additional studies investigating whether primary cilia are required for Gα_S_ coupling and ADCY3 activation by MC4R may be necessary. Moreover, since the primary cilia of MC4R-positive neurons are not solely limited to the PVN, it is necessary to observe the functions of cilia in other MC4R-positive neurons.

### The importance of primary cilia in the ventromedial hypothalamus

It has been reported that *Adcy3* gene polymorphisms are associated with obesity and are known to be exclusively expressed in neuronal cilia [57, 76]. Selective ablation of *Adcy3* by injection of AAV-Cre into the VMH of *Adcy3*-floxed mice significantly increased body fat and led to obesity, supporting the idea that *Adcy3* in the VMH plays an important role in the regulation of energy balance [77].

Recently, we addressed the homeostatic roles of VMH-expressing primary cilia in our research. We deleted VMH-primary cilia by targeting the *Ift88* gene using either steroidogenic factor 1 cre (SF1-Cre) or bilateral AAV-Cre injection and then monitored the metabolic changes [41]. The VMH-specific primary cilia KO (IFT88-KO^SF-1^) mice exhibited metabolic dysregulation linked to decreased sympathetic nervous activity (SNA) and central leptin resistance, which led to marked obesity. The obese phenotype of the IFT88 KO^SF-1^ mice presented with decreased energy expenditure, which appeared to be a primary consequence of reduced sympathetic outflow rather than a secondary effect of obesity. In addition to the energy balance disturbance in the IFT88-KO^SF-1^ mice, VMH-primary cilia have also been shown to be associated with bone density maintenance, suggesting that the altered sympathetic activity induced by deleting VMH-primary cilia might be critical for changing bone density [41]. Further studies delineating how VMH-primary cilia control SNA activity are necessary.

### Leptin signaling and primary cilia in the hypothalamus

The most severe obesity phenotype in humans and mice results from a deficiency of either the leptin or the leptin receptor. Leptin deficiency-associated obesity is resolved upon treatment with recombinant leptin, strongly indicating the critical physiological roles of leptin and leptin signaling in the control of body energy homeostasis [78, 79]. One of the distinct phenotypes of ciliopathy is elevated leptin levels, indicating that leptin resistance may either be a cause or be a consequence of obesity [80, 81]. Thus, the relationship between leptin action and the functional involvement of primary cilia has been investigated.

A potential molecular mechanism of cilia in leptin action was suggested by scientists studying BBS-mutant mice [61]. BBS-mutant mice exhibited hyperphagia and higher leptin levels. Interestingly, they did not respond properly to leptin even after leptin levels were normalized by caloric restriction. Considering these results, Seo et al. suggested that the leptin-resistant phenotype in BBS-mutant mice may be a primary effect of cilia dysfunction [61]. However, Berbari et al. analyzed the leptin response in pre-obese, obese, and food-restricted mice after generating inducible *Ift88* and *Bbs4* KOs [82] and found that the mutant mice showed leptin resistance only under obesity-inducing conditions, strongly indicating that leptin resistance is a secondary consequence of obesity.

In another study, Guo et al. tested leptin sensitivity in LRb^Cre^/Bbs1^fl/fl^ mice that were at comparable body weight with WT littermates, but the response was substantially attenuated [62]. In addition, the LRb^Cre^/Bbs1^fl/fl^ mice under calorie-restricted conditions remained at a higher body weight and fat mass with lower energy expenditure, possibly due to reduction in leptin sensitivity and impaired leptin signaling. In addition to these experiments, the deletion of *Ift88* in SF-1 neurons of the VMH also indicated the involvement of primary cilia in leptin action [41]. We measured leptin levels when the body weights of WT and IFT88-KO^SF-1^ mice were comparable, simultaneously and directly injecting leptin and examining the leptin sensitivity in the WT and IFT88-KO^SF-1^ mice. The physiological response to leptin in the IFT88-KO^SF-1^ mice, portrayed as significantly increased rebound food intake, as well as the effects of an increase in energy expenditure, were blunted, indicating that the deletion of primary cilia in the VMH blunted leptin sensitivity [41].

Taken together, the current data imply that the impact of primary cilia on leptin action may differ among neuron types. To rationalize the molecular mechanism underlying between primary cilia and leptin in energy homeostasis, further observations should be considered to determine (i) whether the leptin receptor exists in the primary cilium and (ii) distinct neuronal populations linking leptin action and the role of primary cilia.

## Conclusions

**Fig. 3:**
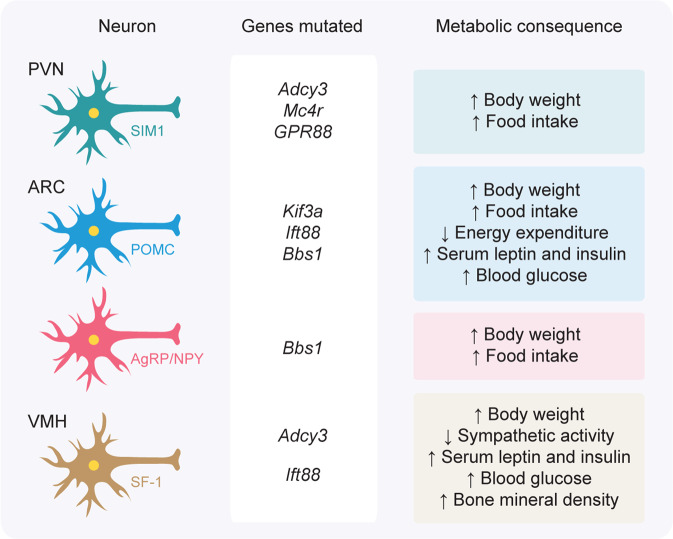
Ciliary genes in the hypothalamic nuclei involved in metabolic dysfunction. Simplified overview of metabolic changes as a consequence of the mutation of ciliary genes in the indicated hypothalamic neurons. Notably, primary cilia play a distinct homeostatic role in each hypothalamic nucleus.

The regulation of energy balance is complex and governed by diverse neuronal factors. Several recent studies have revealed the distinct roles of hypothalamic primary cilia in controlling energy balance (Fig. 3) [41, 61, 62, 64, 77]. Although the functional importance of cilia has been determined, studies are needed to further reveal and to gain full understanding of the molecular composition of neuronal cilia and their precise roles in modulating energy homeostasis, primarily by determining the distinct roles that hypothalamic primary cilia play in different neurons. Moreover, given that cilia biogenic genes such as *Ift88* have cilia-independent roles in tissue development, direct evidence that defects in ciliary formation per se are necessary for acquisition of a pathological phenotype is still needed. Additionally, since primary cilia have been known to contribute to neural development, it would be reasonable to examine whether neuronal cilia influence the formation of the neuronal circuit [64, 83, 84]. In summary, studies of hypothalamic primary cilia offer great potential to gain understanding of other aspects of energy homeostasis regulation through the central nervous system and possibly provide a new strategy to overcome metabolic disorders.
